# LianXia Formula Granule Attenuates Cardiac Sympathetic Remodeling in Rats with Myocardial Infarction via the NGF/TrKA/PI3K/AKT Signaling Pathway

**DOI:** 10.1155/2021/5536406

**Published:** 2021-06-11

**Authors:** Sai-Sai Li, Nan Kang, Xiang-Lei Li, Jing Yuan, Ruby Ling, Ping Li, Jia-Li Li

**Affiliations:** ^1^Beijing University of Chinese Medicine Third Affiliated Hospital, Beijing 100029, China; ^2^Affiliated Hospital of Jining Medical University, Jining 272029, Shandong, China; ^3^Weifang Medical University, Weifang 261053, Shandong, China

## Abstract

Sympathetic remodeling may cause severe arrhythmia after myocardial infarction (MI). Thus, targeting this process may be an effective strategy for clinical prevention of arrhythmias. LianXia Formula Granule (LXFG) can effectively improve the symptoms of patients with arrhythmia after MI, and modern pharmacological studies have shown that *Coptidis Rhizoma* and *Rhizoma Pinelliae Preparata*, the components of LXFG, have antiarrhythmia effects. Here, we investigated whether LXFG can mitigate sympathetic remodeling and suppress arrhythmia and then elucidated its underlying mechanism of action in rats after MI. Sprague-Dawley (SD) rats that had undergone a myocardial infarction model were randomly divided into 6 groups, namely, sham, model, metoprolol, and LXFG groups, with high, medium, and low dosages. We exposed the animals to 30 days of treatment and then evaluated incidence of arrhythmia and arrhythmia scores *in vivo* using programmed electrical stimulation. Moreover, we determined plasma catecholamines contents via enzyme-linked immunosorbent assay and detected expression of tyrosine hydroxylase (TH) at infarcted border zones via western blot, real-time PCR, and immunohistochemical analyses to assess sympathetic remodeling. Finally, we measured key molecules involved in the NGF/TrKA/PI3K/AKT pathways via western blot and real-time PCR. Compared with the model group, treatment with high dose of LXFG suppressed arrhythmia incidence and arrhythmia scores. In addition, all the LXFG groups significantly decreased protein and mRNA levels of TH, improved the average optical density of TH-positive nerve fibers, and reduced the levels of plasma catecholamines relative to the model group. Meanwhile, expression analysis revealed that key molecules in the NGF/TrKA/PI3K/AKT pathways were downregulated in the LXFG group when compared with model group. Overall, these findings indicate that LXFG suppresses arrhythmia and attenuates sympathetic remodeling in rats after MI. The mechanism is probably regulated by suppression of the NGF/TrKA/PI3K/AKT signaling pathway.

## 1. Introduction

Myocardial infarction (MI) is one of the most serious coronary heart diseases, whose morbidity and mortality rates are gradually increasing [1, 2]. Arrhythmia after MI, especially fatal ventricular arrhythmia (VAs), represents the main cause of death in MI patients [3, 4]. Currently, numerous research efforts are focused on elucidating the underlying mechanism of action and developing prevention strategies for arrhythmia after MI.

Sympathetic remodeling is a key factor in induction of arrhythmia after MI [5]. After MI, sympathetic denervation in the infarcted zone and sympathetic hyperinnervation in the infarcted border zone cause uneven and excessive regeneration of sympathetic nerves, as well as a disturbance to their density and spatial structure, in a phenomenon commonly known as sympathetic remodeling [6, 7]. Functionally, sympathetic remodeling can cause electrophysiological disorders and elevated heterogeneity of noradrenergic transmission, which subsequently exacerbates the risk of VAs and causes sudden cardiac death after MI [8]. Therefore, any intervention that targets sympathetic remodeling-related factors may be a potential strategy for reducing incidence of arrhythmias following MI.

Nerve growth factor (NGF), a member of the neurotrophic factor family, reportedly plays a crucial role in repair of sympathetic nerve injury, as well as survival and differentiation of sympathetic neurons [9]. Elevated NGF release and expression is the main mechanism sympathetic remodeling after MI. Previous studies have shown that NGF binds to the tyrosine kinase receptor (TrKA) and promotes survival signals [10, 11]. Consequently, the NGF/TrKA complex triggers activation of phosphatidylinositol 3 kinase/protein kinase B (PI3K/AKT) [12]. Notably, the PI3K/AKT signaling pathway has been shown to play a critical role in the regulation of cell survival across various systems [13, 14]. In the presence of NGF, inhibition of any part of the PI3K/AKT signaling pathway significantly lowers survival of cultured sympathetic neurons [15]. Additionally, numerous studies have affirmed the pathway's crucial role in survival of sympathetic neurons [16, 17]. In the present study, we hypothesized that the NGF/TrKA/PI3K/AKT signaling pathway may also be playing an important role in sympathetic nerve regeneration and remodeling after MI.

Complementary and alternative medicine is commonly used in the treatment of various chronic diseases worldwide [18, 19]. Some studies [20, 21] have reported that different processed ginseng extracts and specific ginsenosides possess beneficial effects on diabetes, especially type 2 diabetes. An evidence-based review [22] showed that herbal remedies in traditional Persian medicine may effectively treat hemorrhoids and more than half of the reported herbs exhibited anti-inflammatory and analgesic effects. Mistletoe therapy is one of the most frequently prescribed oncological treatments in German speaking countries and up to 77% apply complementary mistletoe therapy in the context of integrative oncological approaches [23, 24]. A randomized double-blind placebo-controlled clinical trial [25] revealed positive effect of topical chamomile oil for mild and moderate carpal tunnel syndrome.

Traditional Chinese Medicine, characterized by multiple targets, pathways, and links, has shown promise in prevention and treatment of arrhythmia after MI [26, 27]. For example, LianXia Formula Granule (LXFG) [28], comprising *Coptidis Rhizoma* and *Rhizoma Pinelliae Preparata*, was shown by Professor Li Ping to manage coronary heart disease with phlegm-heat syndrome. In our previous study, we found that LXFG could reverse ventricular remodeling, improve heart function in rats with MI, and antagonize adrenaline-induced arrhythmia [29, 30]. Since it is not known whether LXFG has beneficial effects on arrhythmia following MI, the present study sought to provide evidence on this topic, by focusing on sympathetic remodeling and the NGF/TrKA/PI3K/AKT signaling pathway.

## 2. Materials and Methods

### 2.1. Ethics Statement

All experimental protocols and animal handling procedures used in this study were approved by the Ethics Committee of Affiliated Hospital of Jining Medical University. Experimental work was performed in accordance with the Guide for the Animal Care and Use of Laboratory Animals published by the National Institutes of Health (NIH Publications No. 85–23, revised 1996). We made every effort to minimize animal suffering and the number of rats used in this study.

### 2.2. Animals

Male Sprague-Dawley rats, weighing between 200 and 220 g, were purchased from Beijing Vital River Laboratory Animal Technology Co., Ltd. (License No. SCXK (Beijing) 2016–0006). The animals were housed under a 12-hour light/dark cycle, with the lights on from 8 am, and allowed free access to normal rat chow and drinking water.

### 2.3. Drugs

LXFG, produced by Beijing Kangrentang Pharmaceutical Co., Ltd., Beijing, China, consisting of 2 Chinese herbs, *Coptidis Rhizoma* (batch number: 17025661) and *Rhizoma Pinelliae Preparata* (batch number: 17022201), was purchased from Beijing University of Chinese Medicine Third Affiliated Hospital. Metoprolol tartrate tablets were produced by AstraZeneca Pharmaceutical Co., Ltd., Jiangsu, China (Med-drug permit number H32025391).

### 2.4. Establishment of MI Rat Model

SD rats were anesthetized with 1% pentobarbital sodium (40 mg/kg), by intraperitoneal injection, under intubation and mechanical ventilation using a small rodent ventilator (Alcott Biotech Co., Ltd., Shanghai, China). A standard limb lead II electrocardiogram was continuously recorded with an ECG recorder (multichannel physiological signal acquisition and processing system, Chengdu, China). Thoracotomy was performed in the left third intercostal space, and the left atrial appendage exposed then ligated directly under the original 2 mm of the left atrial appendage by a 5/0 suture. Thoracic closure was done by suturing the three layers. Successful MI model establishment was confirmed when the color of the anterior wall of left ventricle changed to a pale color, the ventricular wall motion reduced, and electrocardiograph revealed elevation of the ST segment. After surgery, the rats were administered with 400,000 units of penicillin and then placed on an electric blanket until they woke. Rats in the sham group were subjected to steps involving concentric infarction modeling, with no knots placed after threading.

### 2.5. Animal Grouping and Drug Administration

Rats with successful coronary artery ligation were randomly divided into a model, metoprolol, high-dose LXFG (LXFG-H), medium-dose LXFG (LXFG-M), and low-dose LXFG (LXFG-L) groups, with 10 rats in each group. An additional sham group consisted of 8 rats that did not have actual LAD ligation. Drug dosages were administered according to the rat conversion coefficient of human clinical dose in experimental zoology [31] and an adult clinical dose for LXFG at 0.9 g/d. Therefore, rats in the LXFG-H, LXFG-M, and LXFG-L groups were administered with a daily LXFG dose of 189.00 (H), 94.50 (M), and 47.25 mg/kg (L), respectively, dissolved in distilled water, by oral gavage. Rats in the metoprolol group were given 24 mg/kg of metoprolol tartrate tablets, whereas those in the sham and model groups were given an equivalent amount of distilled water. All rats were administrated treatment intragastrically for 30 days, before use in subsequent experiments.

### 2.6. Electrophysiological Study

Programmed electrical stimulation (PES) was performed as previously reported [32, 33]. Briefly, rats were anesthetized, their hearts exposed as described above, and then electrocardiograms continuously recorded with PowerLab multichannel physiological recorder (ADInstruments, New South Wales, Australia). Induction of VAs was performed by ventricular stimulation at a basic cycle length of 120 ms (S1) for eight paced beats, followed by one to two extra stimuli (S2 and S3) at shorter coupling intervals. The end point of PES involved induction of VAs consisting of at least 6 consecutive nondriven ventricular extra stimulus beats. A preparation was considered noninducible if PES produced either no or less than 6 beats. Moreover, ventricular tachyarrhythmias were considered nonsustained and sustained if they lasted for less than and more than 15 beats, respectively. The arrhythmia scores were determined as follows: 0, noninducible; 1, nonsustained tachyarrhythmias induced with three extra stimuli; 2, sustained tachyarrhythmias induced with three extra stimuli; 3, nonsustained tachyarrhythmias induced with two extra stimuli; 4, sustained tachyarrhythmias induced with two extra stimuli; 5, nonsustained tachyarrhythmias induced with one extra stimulus; 6, sustained tachyarrhythmias induced with one extra stimulus; 7, tachyarrhythmias induced during a train of eight stimuli (8 × S1) at a basic cycle length of 120 ms; and 8, heart stopped before programmed electrical stimulation. When multiple forms of arrhythmias occurred in one heart, the highest score was used.

### 2.7. Enzyme-Linked Immunosorbent Assay

Blood samples were collected from the abdominal aorta of all rats, plasma was isolated, and then levels of plasma catecholamines (NE, EPI, and DA) were quantified using a commercial ELISA kit (Elabscience Biotechnology Co., Ltd., Wuhan, China), according to the manufacturer's instructions. Absorbance was measured at 450 nm, using a microplate reader, and concentrations in the samples were calculated relative to corresponding standard curves.

### 2.8. Immunohistochemical Staining

We investigated TH localization and distribution via immunohistochemical staining. Briefly, the infarct border zone of the left ventricular heart tissue was collected and embedded in paraffin, and 5 µm thick sections were cut using a microtome. The sections were dewaxed, hydrated, and antigen repaired, with a 15-minute incubation at room temperature (RT) in 3% hydrogen peroxide. After blocking them with goat serum for 30 min at RT, the sections were incubated overnight with a rabbit polyclonal anti-tyrosine hydroxylase antibody (1 : 200, Abcam, UK) at 4°C and then with HRP-conjugated goat anti-rabbit IgG antibody for 30 min at RT. They were then stained with DAB solution and hematoxylin, according to the manufacturer's instructions. The slides were dehydrated, cleared with xylene, mounted with permanent mounting medium, and photographed. Resulting images were analyzed using IPP 6.0 software, with six random fields of each slide selected, and used to calculate average optical density (AOD) of the TH-positive nerve fibers.

### 2.9. Western Blot Analysis

Samples of myocardial tissues from the infarcted border zone were prepared for protein analysis. The heart tissues samples were homogenized in RIPA lysis buffer containing a protease inhibitor cocktail. Protein concentrations were determined using the BCA kit (Beyotime, Shanghai, China), and 40 *μ*g protein lysates were electrophoresed and separated on 10–15% SDS-PAGE before transfer onto PVDF membranes (Millipore, Boston, USA). The membranes were blocked with 5% skim milk for an hour at room temperature and then incubated overnight at 4°C with primary antibodies, namely, rabbit anti-NGF (1 : 1000, Abcam, UK), rabbit anti-TrKA (1 : 1000, Abcam, UK), rabbit anti-tyrosine hydroxylase (1 : 10000, Abcam, UK), rabbit anti-p-PI3K (1 : 1000, affinity, USA), rabbit anti-p-Akt (1 : 1000, Cell Signaling Technology, USA), and rabbit anti-p-Bad (1 : 1000, ImmunoWay, USA). The membranes were washed thrice with Tris-Buffered Saline Tween, followed by incubation with the horseradish peroxidase-conjugated secondary antibody (1 : 5000) for 2 hours at RT. Antigen-antibody complexes were then visualized using an enhanced chemiluminescence kit (Applygen, Beijing, China), and relative band densities of proteins in the western blots were normalized against GAPDH.

### 2.10. Quantitative Real‐Time Polymerase Chain Reaction (qRT-PCR)

Total RNA was isolated from the infarcted border zone of rat heart using the TRIzol reagent (Invitrogen, California, USA) and subjected to complementary DNA (cDNA) synthesis using the RevertAid First Strand cDNA Synthesis Kit (Invitrogen, California, USA). qRT-PCR was performed using the SYBR Green Master Mix (Vazyme) on an ABI QuantStudio 6 PCR instrument (Applied Biosystems, New York, USA), under the following conditions: denaturation, followed by 40 cycles of 50°C for 2 min, 95°C for 10 min, 95°C for 30 s, and 60°C for 30 s). mRNA expression levels were normalized to those of GAPDH and calculated using the 2^−ΔΔ*Ct*^ method. The primers used in the study are listed in Table 1.

### 2.11. Statistical Analysis

Statistical analyses were performed using SPSS 25.0 (IBM, Armonk, USA). Quantitative data were presented as means ± standard deviations (SD). Differences among groups were determined using a one-way analysis of variance (ANOVA), whereas arrhythmia scores were examined by Kruskal–Wallis test. Statistical significance was set at *P* < 0.05.

## 3. Result

### 3.1. LXFG Reduced the Arrhythmia Susceptibility

**Figure 1:**
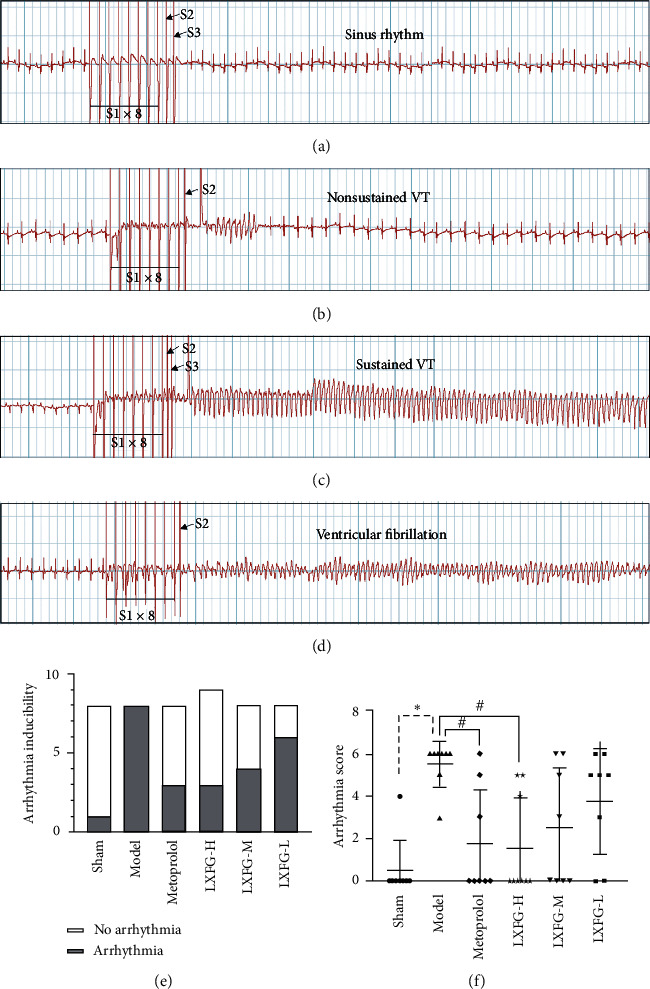
LXFG reduced arrhythmia susceptibility. Representative ECG of electrical stimulation, including (a) sinus rhythm, (b) nonsustained ventricular tachycardia, (c) sustained ventricular tachycardia, and (d) ventricular fibrillation. (e) Inducibility of arrhythmia after MI. (f) Inducible arrhythmia scores. *n* = 9 and *n* = 8 for rats in the LXFG-H and other groups, respectively. ^*∗*^*P* < 0.05 versus sham group; ^#^*P* < 0.05 versus model group.

We performed PES to confirm the beneficial effect of LXFG on arrhythmia following MI. No rats experienced spontaneous VAs during the placement of the electrodes, and none of them died during the electrophysiological study. Evaluation of ventricular arrhythmia severity, by arrhythmia inducibility and arrhythmia score, revealed an induction rate of 12.5% (1/8) and an arrhythmia score of 0.75 ± 2.12 in sham group. Arrhythmia incidence and arrhythmia score in the model group were 100% (8/8) and 5.13 ± 1.81, respectively, and represented a significant increase compared with the sham group (*P* < 0.05). On the other hand, VAs induction rates were lower for rats in the LXFG-H, LXFG-M, and LXFG-L groups, recording 33.3% (3/9), 50% (4/8), and 75% (6/8), respectively (Figure 1(e)). Arrhythmia scores for rats in the LXFG group were lower than those in the model group and showed a dose-dependent decline. Moreover, those in the LXFG-H were significantly different from the model group (*P* < 0.05, Figure 1(f)), suggesting that LXFG could reduce susceptibility to VAs after MI.

### 3.2. LXFG Attenuated Sympathetic Nerve Remodeling

TH refers to the rate-limiting enzyme of norepinephrine synthesis and serves not only as a marker for sympathetic nerve location but also as an indicator of sympathetic activity [3, 34, 35]. To determine the effect of LXFG inhibition on sympathetic neural remodeling, we assessed TH expression in the infarcted border zone of rat cardiac tissues.

**Figure 2:**
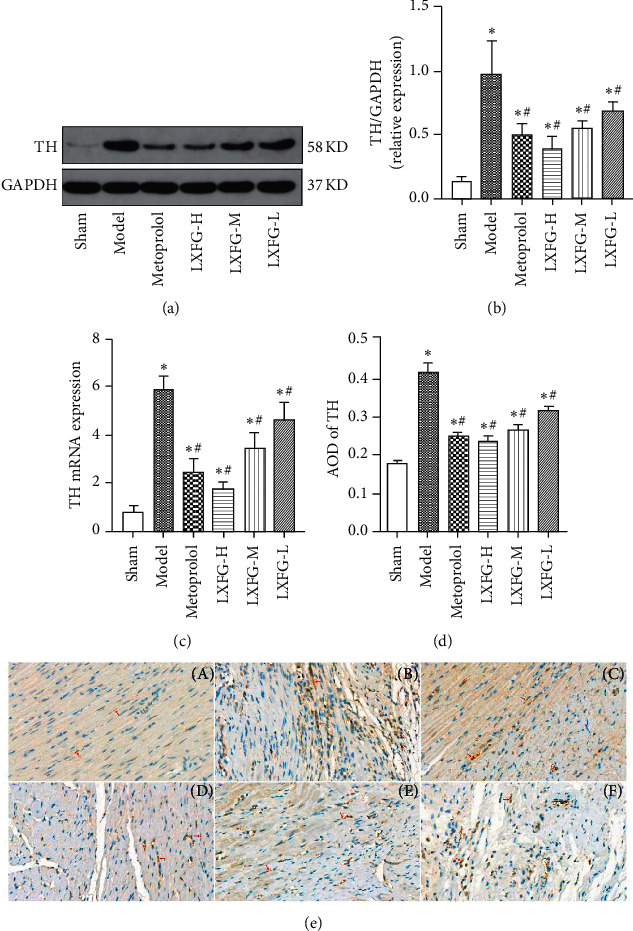
Effect of LXFG on sympathetic remodeling in the infarcted border zone after MI. (a) Western blots for TH and GAPDH in heart. (b) Profiles of relative protein expression of TH in each group (*n* = 4). (c) Profiles of relative mRNA expression of TH in each group (*n* = 6). (d) Quantitative analysis of TH-positive nerve fibers (*n* = 6). (e) Immunohistochemical staining for TH. (a) Sham group. (b) Model group. (c) Metoprolol group. (d) LXFG-H group. (e) LXFG-M group. (f) LXFG-L group. Scale bar = 20 *μ*m. ^*∗*^*P* < 0.05 versus sham group; ^#^*P* < 0.05 versus model group.

Profiles of protein and mRNA expression of TH indicated significantly elevated levels in the model compared to the sham group (*P* < 0.05). However, exposure to different LXFG dosages significantly reduced levels of both protein and mRNA expression of TH in LXFG and metoprolol groups, relative to the model group (*P* < 0.05, Figures 2(a)–2(c)).

In the immunohistochemical staining, the TH-positive nerve fibers are marked brown, located between the myofibrils and arranged longitudinally. There were few TH-positive nerve fibers in the sham group, which were consistent with the direction of myocardial cells. In the model group, the expression of TH was significantly increased, and the morphology was thick and disorderly, and some of them were aggregated into bundles. Immunohistochemical staining analyses validated cardiac sympathetic remodeling in model rats, while LXFG treatment reversed this effect (Figures 2(d)–2(e)). Specifically, the average optical density of TH-positive nerve fibers in the infarcted border zone was significantly higher in the model group (0.41 ± 0.02) than in the sham group (0.18 ± 0.01) (*P* < 0.05). However, this increase was significantly lower across different LXFG groups (LXFG-H: 0.24 ± 0.01, LXFG-M: 0.26 ± 0.02, and LXFG-L: 0.30 ± 0.01) and metoprolol group (0.26 ± 0.01) relative to the model group (*P* < 0.05).

### 3.3. LXFG Suppressed Plasma Catecholamines Levels

**Figure 3:**
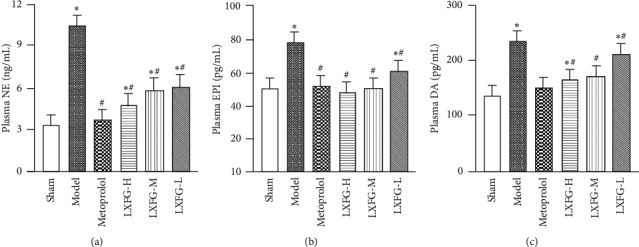
Effect of LXFG on plasma catecholamines. *n* = 9 and *n* = 8 rats in LXFG-H and other groups, respectively. ^*∗*^*P* < 0.05 versus sham group; ^#^*P* < 0.05 versus model group.

We evaluated peripheral sympathetic activity by determining levels of plasma catecholamines, including norepinephrine (NE), epinephrine (EPI), and dopamine (DA). Results revealed significant upregulation of these in the model, relative to the sham group (*P* < 0.05) (Figure 3). Moreover, rats in different LXFG and metoprolol groups exhibited significantly lower plasma catecholamine levels than those in the model group (*P* < 0.05).

### 3.4. LXFG Affected mRNA Expression in the NGF/TrKA/PI3K/AKT Signaling Pathway

**Figure 4:**
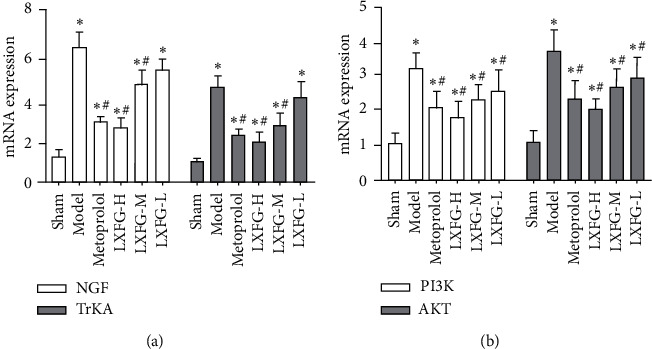
Profiles of mRNA expression of the NGF/TrKA/PI3K/AKT signaling pathway in the infarcted border zone in rats in each group (*n* = 6). ^*∗*^*P* < 0.05 versus sham group; ^#^*P* < 0.05 versus model group.

Levels of NGF, TrKA, PI3K, and AKT mRNAs were significantly upregulated in the model (*P* < 0.05), relative to the sham group (Figure 4). Conversely, rats in the LXFG-H, LXFG-M, and metoprolol groups exhibited significantly downregulated NGF, TrKA, PI3K, and AKT mRNA levels (*P* < 0.05). Moreover, LXFG-L only downregulated mRNA expression of PI3K and AKT (*P* < 0.05), although it had no statistically significant differences on NGF and TrKA (*P* > 0.05).

### 3.5. LXFG Affected Expression of Key Proteins in the NGF/TrKA/PI3K/AKT Signaling Pathway

**Figure 5:**
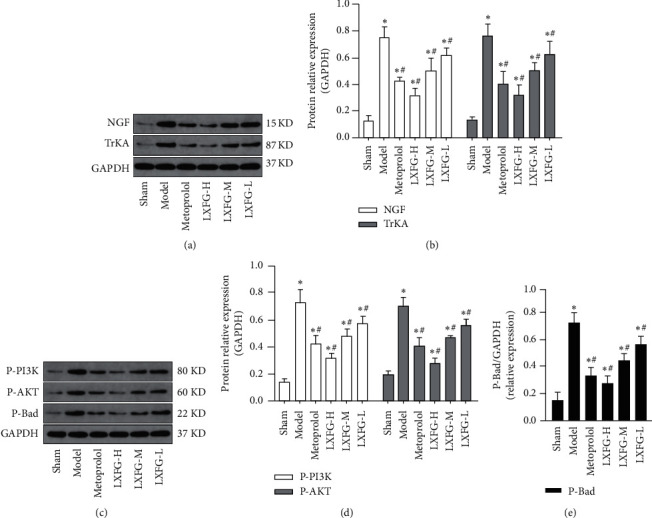
Expression profiles of key proteins in the NGF/TrKA/PI3K/AKT signaling pathway in the infarcted border zone in rats in each group (*n* = 4). ^*∗*^*P* < 0.05 versus sham group; ^#^*P* < 0.05 versus model group.

To clarify the action mechanism of LXFG, we used western blot to analyze expression levels of proteins in the NGF/TrKA/PI3K/AKT signaling pathway. Since phosphorylated proteins can regulate intracellular signal transduction, whereas P-Bad is a downstream factor of the PI3K/AKT signaling pathway mediating survival, we specifically detected P-PI3K, P-AKT, and P-Bad proteins. Western blots revealed significant upregulation of NGF, TrKA, P-PI3K, P-AKT, and P-Bad in the model, relative to the sham group (*P* < 0.05) (Figure 5). On the contrary, exposure to LXFG (LXFG-H, LXFG-M, and LXFG-L) and metoprolol resulted in significant downregulation of NGF, TrKA, P-PI3K, P-AKT, and P-Bad proteins, relative to the model group (*P* < 0.05). Overall, these findings indicated that LXFG suppresses expression of the NGF/TrKA/PI3K/AKT signaling pathway.

## 4. Discussion

Our results revealed significant upregulation of the NGF/TrKA/PI3K/AKT signaling pathway after MI. Moreover, LXFG treatment suppressed arrhythmia, alleviated sympathetic remodeling in the infarcted border zone, and suppressed overactivation of the NGF/TrKA/PI3K/AKT signaling pathway after MI.

MI induction initiates a dynamic evolution process of degeneration, necrosis, regeneration, and remodeling of cardiac sympathetic nerve. Sympathetic remodeling, a repair response to ischemic injury, has been hypothesized to be a protective mechanism [36]. However, excessive nerve regeneration and remodeling is harmful to the body, especially in the infarcted border zone, where sympathetic remodeling is very active [4, 37]. Overactivation of cardiac sympathetic nerve activity significantly elevates abnormal excitability, automaticity, and conductivity of residual cardiac myocytes, leading to quickly emitting impulses, thereby initiating arrhythmia [38]. Furthermore, previous studies have shown that a large number of secreted catecholamines may directly or indirectly affect cardiac electromechanical and nerve activity, aggravate instability of the electrocardiogram, and cause various types of arrhythmia [6]. Cardiac sympathetic remodeling represents an important pathological basis of arrhythmia after MI.

Results of the present study revealed significantly higher incidence of arrhythmia and arrhythmia scores in the model group, relative to the sham group. Moreover, we observed significantly elevated expression of TH proteins and mRNAs in the infarcted border zone, while TH-positive nerve fibers were disordered, indicating that sympathetic remodeling increases the susceptibility to arrhythmia after MI. However, incidence of arrhythmia after MI exhibited a dose-dependent decline after 30 days of LXFG treatment. In addition, LXFG treatment significantly alleviated sympathetic remodeling, indicating a harmonious distribution of the sympathetic nerve. Overall, these findings suggested that LXFG may be a suitable and effective alternative medicine for treating arrhythmia following MI.

To elucidate the molecular mechanisms underlying LXFG's attenuation of sympathetic remodeling in rats, we targeted the NGF/TrKA/PI3K/AKT signaling pathway. Previous studies have shown that NGF plays a pivotal role in regulating neuronal survival, plasticity, and disease [39], with expression of cardiac NGF found to be positively correlated with the density of cardiac sympathetic nerves [40]. Previous studies have demonstrated that transgenic mice overexpressing NGF in the heart showed cardiac hyperinnervation [41], whereas the volume of sympathetic ganglia was significantly reduced in NGF knockout mice [42]. Taken together, these studies affirm NGF's crucial role in post-MI sympathetic sprouting. Functionally, NGF exerts its neurotrophic effect by interacting with a high-affinity receptor, TrKA. The PI3K/AKT participates in the NGF/TrKA signal transduction cascade, thereby regulating neuron survival [43]. A previous study showed that PI3K's catalytic subunit secretes PIP3 on the inner surface of the cytoplasmic membrane of the cell, which subsequently acts as the second messenger in the cell and combines with the PH domain of AKT, to cause conformational changes promote AKT activation [44]. Moreover, Ginty et al. [45] demonstrated that a functional PI3K/AKT pathway is required for neurotrophins to promote neuronal survival both inside the cell body and in the distal axons, while Singh et al. [46] found that PI3K/AKT could further regulate its downstream factors to transduce survival effects. Activated Akt phosphorylates Ser136 residues of the Bad protein, a proapoptotic protein of the Bcl-2 family, found downstream of AKT [47]. Phosphorylated Bad dissociates from the apoptosis-promoting complex and forms a 14-3-3 protein complex, which inactivates its apoptosis-promoting function and exerts antiapoptotic effect [48]. Taken together, the NGF/TrKA/PI3K/AKT signaling pathway represents an important target for attenuating sympathetic remodeling after MI.

**Figure 6:**
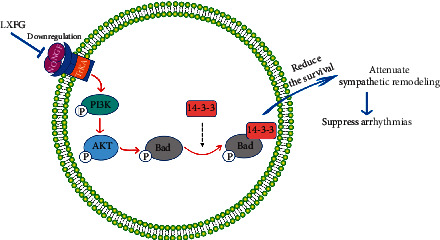
LianXia Formula Granule alleviates arrhythmia in rats with myocardial infarction via the NGF/TrKA/PI3K/AKT signaling pathway. LianXia Formula Granule treatment downregulated the expression of key molecular proteins and mRNA of NGF/TrKA/PI3K/AKT signaling pathway as well as P-Bad; attenuated sympathetic remodeling in the infarcted border zone of rat cardiac tissues. LXFG, LianXia Formula Granule; NGF, nerve growth factor; TrKA, tyrosine kinase receptor A; PI3K, phosphatidylinositol 3-kinase; AKT, protein kinase B.

In this study, we successfully established a MI model by ligating rats' left anterior descending coronary artery. Western blots and qRT-PCR results revealed significant upregulation of key proteins and mRNAs in the NGF/TrKA/PI3K/AKT signaling pathway as well as P-BAD in the model group. These results suggested that sympathetic remodeling in the model group might be associated with increased activation of the NGF/TrKA/PI3K/AKT signaling pathway, which mediates excessive sympathetic survival in the infarcted border zone. LXFG's ability to downregulate expression of key molecular proteins and mRNA of the NGF/TrKA/PI3K/AKT signaling pathway indicated that this drug has inhibitory effects on the pathway (Figure 6). Therefore, we speculated that LXFG may be regulating expression of effector P-Bad via the NGF/TrKA/PI3K/AKT signaling pathway, causing a balance between survival, regeneration, and apoptosis of sympathetic neurons, thereby attenuating sympathetic remodeling and reducing occurrence of arrhythmia in rats following MI.

TCM has been used, for more than 2,000 years, to treat cardiovascular diseases as well as for prevention and treatment of myocardial infarction diseases [49]. Currently, TCM is increasingly becoming a popular alternative or complementary medicine across many countries due to the urgent need for multitarget, multipathway, and personalized treatment [50]. One such TCM, LXFG, has shown efficacy in clearing heat and eliminating phlegm, as well as calming the heart and relieving palpitations. Modern pharmacological studies have revealed that Berberine is the most important active ingredient of *Coptidis Rhizoma* and confers several cardiovascular protective effects, including lowering total blood cholesterol, alleviating arrhythmia, and improving myocardial infarction and injury in cardiomyocytes [51, 52]. A previous study also showed that Berberine-based preconditioning significantly prevented myocardial I/R injury, alleviated ventricular arrhythmia, and suppressed inflammatory infiltration by inhibiting activation of the PI3K/AKT signaling pathway [14]. Previous studies have shown that active ingredients of *Rhizoma Pinelliae Preparata* play a critical role in treatment of cardiovascular system diseases, where they suppress oxidative stress, repress inflammatory cascade, and lower blood pressure [53, 54]. In addition, *Rhizoma Pinelliae* can regulate SOD, MDA, and SA-*β*-gal levels via the PI3K/AKT signaling pathway, thereby delaying senescence and treating atherosclerosis [55]. These ingredients may be the underlying factors regulating LXFG's cardioprotective effects after MI.

This study had some limitations. Firstly, we did not consider inhibitors of the NGF/TrKA/PI3K/AKT signaling pathway. Secondly, we did not test LXFG's effect using cell experiments. Therefore, future studies, using a combination of a variety of inhibitors and detection methods, are needed to validate exact regulatory targets and underlying mechanisms of LXFG.

## 5. Conclusion

In summary, LXFG reduces arrhythmia and attenuates sympathetic remodeling after MI, by suppressing expressions genes and proteins in the NGF/TrKA/PI3K/AKT signaling pathway. High dose of LXFG has a more significant effect over low dose and medium dose. Our findings have critical clinical implications and provide novel insights to guide development of therapies for prevention and treatment of arrhythmia following MI.

## Figures and Tables

**Table 1:** Primer sequences used in this experiment.

Primer	Forward sequence (5′-3′)	Reverse sequence (5′-3′)
TH	GAGCCTTTGACCCAGACACA	GGGCTGTCCAGTACGTCAAT
NGF	AGACCCGCAACATCACTG	CGTGGCTGTGGTCTTATCTC
TrKA	GACCTCAACCGTTTCCTCC	CATGCCGAAGTCTCCAATCT
PI3K	GCAACAAGTCCTCTGCCAAA	ACGTAATAGAGGAGCTGGGC
AKT	TTTATTGGCTACAAGGAACG	GGTGTAGTTCAGAGGCAGGT
GAPDH	ACAGCAACAGGGTGGTGGAC	TTTGAGGGTGCAGCGAACTT

## Data Availability

The data used to support the findings of this study are available from the corresponding author upon reasonable request.
